# Eliminating health care inequities through strengthening access to care

**DOI:** 10.1111/1475-6773.14202

**Published:** 2023-11-28

**Authors:** Monique Jindal, Krisda H. Chaiyachati, Vicki Fung, Spero M. Manson, Karoline Mortensen

**Affiliations:** ^1^ Department of Academic Internal Medicine University of Illinois Chicago Chicago Illinois USA; ^2^ Verily, Inc. South San Francisco California USA; ^3^ Perelman School of Medicine at the University of Pennsylvania Philadelphia Pennsylvania USA; ^4^ Department of Medicine, Harvard Medical School, Mongan Institute Massachusetts General Hospital Boston Massachusetts USA; ^5^ Centers for American Indian and Alaska Native Health University of Colorado Anschutz Medical Campus Aurora Colorado USA; ^6^ Department of Health Management and Policy Miami Herbert Business School Coral Gables Florida USA

**Keywords:** health disparities, health services research, health care access, inequities, intersectionality, racially/ethnically minoritized populations, structural racism

## Abstract

**Objective:**

To provide a research agenda and recommendations to address inequities in access to health care.

**Data Sources and Study Setting:**

The Agency for Healthcare Research and Quality (AHRQ) organized a Health Equity Summit in July 2022 to evaluate what equity in access to health care means in the context of AHRQ's mission and health care delivery implementation portfolio. The findings are a result of this Summit, and subsequent convenings of experts on access and equity from academia, industry, and the government.

**Study Design:**

Multi‐stakeholder input from AHRQ's Health Equity Summit, author consensus on a framework and key knowledge gaps, and summary of evidence from the supporting literature in the context of the framework ensure comprehensive recommendations.

**Data Collection/Extraction Methods:**

Through a stakeholder‐engaged process, themes were developed to conceptualize access with a lens toward health equity. A working group researched the most appropriate framework for access to care to classify limitations identified during the Summit and develop recommendations supported by research in the context of the framework. This strategy was intentional, as the literature on inequities in access to care may itself be biased.

**Principal Findings:**

The Levesque et al. framework, which incorporates multiple dimensions of access (approachability, acceptability, availability, accommodation, affordability, and appropriateness), is the backdrop for framing research priorities for AHRQ. However, addressing inequities in access cannot be done without considering the roles of racism and intersectionality. Recommendations include funding research that not only measures racism within health care but also tests burgeoning anti‐racist practices (e.g., co‐production, provider training, holistic review, discrimination reporting, etc.), acting as a convener and thought leader in synthesizing best practices to mitigate racism, and forging the path forward for research on equity and access.

**Conclusions:**

AHRQ is well‐positioned to develop an action plan, strategically fund it, and convene stakeholders across the health care spectrum to employ these recommendations.

**What is known on this topic**

- Inequities in health care access persist, particularly for racially/ethnically minoritized populations and economically, socially, and geographically disadvantaged populations.
- Racism and bias have exacerbated access issues and resulted in inequities in access to care.
- AHRQ has successfully funded research that has informed all dimensions of access to care with significant success, but it does not integrate a health equity lens across its funding or delivery implementation portfolio.

**What this study adds**

- Expert consensus and evaluation of the literature provide recommendations for AHRQ to serve as the leading organization addressing the pervasiveness of racism in access to care while integrating intersectionality.
- AHRQ can leverage partnerships to enhance the work being done across public and private entities to address multiple aspects of access to care, such as system trustworthiness, community engagement, health literacy, provider competency, workforce diversity, provider shortages, insurance disparities, and patient‐centered tools that address both racism and intersectionality.
- AHRQ can address inequities in access to care by collaboratively funding research that uses a health equity lens to address key gaps in the five dimensions of health care access.

## 1 INTRODUCTION

Access to health care, one of the five pillars of the Agency for Healthcare Research and Quality's (AHRQ) health equity framework,^1^ plays a critical role in improving the health of a population. Access is “the opportunity to identify health care needs, to seek health care services, to reach, to obtain, or to use health care services, and to actually have a need for services fulfilled.”^2^ This working definition of access is within the scope of AHRQ's focus on quality and research. While all populations are relevant to the discussion of access to care, widespread inequities in access to care disproportionately exist for racially/ethnically minoritized populations and economically, geographically, and socially disadvantaged groups. These inequities are exacerbated when considering an individual's or population's intersectionality (i.e., the overlapping experiences of discrimination for people who identify with multiple social dimensions such as race/ethnicity, sex, disabilities, and sexual and gender minorities, which include lesbian, gay, bisexual, transgender, and gender‐nonbinary or gender‐nonconforming individuals).^3^, ^4^ This highlights the importance of examining root causes of such inequities, including marginalization, systemic oppression, and racism, and necessitates the examination of health care access through the lens of health equity.^5^, ^6^, ^7^, ^8^, ^9^, ^10^, ^11^, ^12^, ^13^, ^14^, ^15^, ^16^, ^17^, ^18^, ^19^, ^20^, ^21^

Health equity is the state in which there is “*a fair and just opportunity for everyone to attain their highest level of health*.”^14^ Health equity, however, continues to be threatened by the historical and contemporary injustices that create obstacles to health care access and thereby reinforce health inequities.^14^ Such structural barriers to health equity can include a low density of health care providers in underserved areas,^22^ decreasing primary care density in rural areas,^22^, ^23^ poor transportation infrastructure,^24^, ^25^ lack of adequate broadband high‐speed internet access,^26^ and gaps in insurance coverage, which exacerbate inequitable access to care.^12^, ^27^, ^28^, ^29^

Concrete examples of inequities resulting from these barriers are evident across the access spectrum. Racially/ethnically minoritized populations are more likely to be uninsured; 14% of Black, 25% of Hispanic, and 24% of American Indian/Alaska Native (AI/AN) adults are without insurance, whereas only 8% of White, non‐elderly adults are uninsured.^30^ Half (52%) of White adults with mental illness received mental health services in 2021, compared to 39% of Black, 36% of Hispanic, and 25% of Asian adults.^31^ Hispanic adults were more likely to be without a personal health care provider (34%), as were Black adults (18%) and AI/AN adults (24%), relative to White adults (16%).^31^ Minoritized populations are more likely to not see a doctor due to cost (18% of Hispanic adults, 15% of AI/AN adults, and 14% of Black adults) than Whites (9%).^31^ These access inequities, among others, consequently contribute to overall health inequities^13^ including delayed receipt of preventative services,^32^ higher maternal mortality,^33^ differential treatment outcomes,^13^, ^34^ and premature mortality from conditions that are treatable with timely access to high‐quality care.^12^, ^35^

## 2 METHODS

Acknowledging the importance of health care access' contribution to health equity, AHRQ created an *access* workgroup as part of its Health Equity Summit held in July 2022. The structure and proceedings of the summit are summarized elsewhere (Appendix S1). The goal of AHRQ's Summit was to build consensus on how health equity can be achieved in the context of health systems and in consideration of AHRQ's mission and health care delivery implementation portfolio. Mistry, et al.^1^ overview the key highlights of the Summit in this special issue.

Following the Summit, a writing team reviewed the findings from the workgroup discussion. The themes identified by the Summit workgroup were discussed, as were numerous frameworks for health care access that could be used as an organizational structure for the manuscript. The Summit discussion highlighted limitations of existing frameworks, lack of clarity of some constructs, and need for consideration of both systemic and patient‐side factors.

Through a review of access to care frameworks in the literature, the team reached consensus on utilizing the framework by Levesque, Harris, and Russell.^2^ The framework addresses many of the limitations noted by workgroup members and provides the backdrop for a closer examination of the intersection of health equity and access to care. The model illustrates how access occurs along a continuum and is dependent on five dimensions: approachability, acceptability, availability and accommodation, affordability, and appropriateness.^2^, ^36^ The model conceptualizes access by integrating demand‐side (e.g., predisposing factors, knowledge, and attitudes)^37^ and supply‐side (e.g., location, effective availability, and cost)^38^ factors and how they are influenced by the health system and the patient's perspective. Overall, the model fosters a balanced approach to support our goal of identifying necessary gaps AHRQ must overcome to help advance achieving health equity.^2^

Using the Levesque model, we build on stakeholder feedback from the Summit regarding priority areas across the access to care continuum using a health equity lens. Prioritizing themes identified during the Summit, we describe the current state of the literature and provide recommendations for AHRQ, using their core competencies—health services research, improvement science, data and analytics, and training—to move these fields forward and promote health equity (Table 1). The essential theme is to encourage AHRQ to leverage partnerships with other federal agencies and develop public‐private partnerships in pursuit of health equity. The sections within the manuscript are organized by Levesque et al.'s five dimensions of access.^2^ The referenced literature is an in‐depth exploration of timely issues in health equity and access to care to develop recommendations that best reflect AHRQ's strengths and the workgroup's priority concerns for research, rather than a broader systematic review of the literature. While we do not detail how all marginalized populations are impacted by health inequities, our recommendations should not be seen as weighting one group more important than another. We are resolute in our belief that *all* systemically oppressed groups and populations should have equitable access to health care.

**TABLE 1:** Access to care with health equity lens: challenges and opportunities.

Access dimension	Definition	System‐level barriers to achieving equity	Recommendations
Approachability	The awareness of populations with health needs that services exist, can be reached, and will have an impact on their health	Lack of trustworthiness of institutions and systems	Collaborate to develop and evaluate an evidence‐based, trustworthy anti‐racist toolkit for systems.
		Lack of patient and community input into strategies to address approachability barriers	Provide training in co‐production and community‐engaged research to promote novel information dissemination strategies
		Health literacy occurs after interaction with the health care system	Fund innovative research on addressing health literacy before interactions with the health care system
Acceptability	The intersection of the cultural and social factors associated with a health system and patients' perceptions regarding the extent to which these factors and, in turn, the services provided may meet their needs and preferences	Structural bias rooted in systems and institutions that results in health inequities	Serve as the convening agency for championing use of evidence‐based tools that combat racism Develop and fund evidence‐based curriculum for providers and funders
		Lack of racial/ethnic diversity of workforce	Widely implement holistic review in recruitment; bolster retention efforts; fund research of pipeline programs with longer horizons
Availability and accommodation	The physical existence of health resources with sufficient capacity that can be reached in a timely manner	Provider and physician shortages	Fund collaborative research to identify causes of burnout with a lens toward intersectionality and how mistreatment perpetuates burnout
Affordability	The relationship between direct, indirect, and opportunity costs associated with obtaining services and patients' ability to pay	Insurance coverage disparities and cost sharing	Incentivize public‐private partnerships to develop unified standards of race and ethnicity measurement and assess experiences of racism and discrimination while accessing insurance
		Enrolling and disenrolling in insurance (churn) and gaps in the Affordable Care Act coverage provisions	Fund research to inform states and the federal government on the additional costs of state Medicaid programs in maintaining the flexibility offered in the PHE and identify coverage improvements to the ACA
Appropriateness	The fit between services and needs of the patient, community, and population	Low‐value care and race‐based algorithms impact minority patients Lack of patient‐centered clinical decision support tools informed by intersectionality	Fund research on de‐implementation of low‐value care and develop alternatives to race‐based algorithms Fund cutting edge research on patient‐centered decision‐making tools that integrate intersectionality with a health equity lens

## 3 APPROACHABILITY

Health care is considered *approachable* in the Levesque et al. framework when populations with health needs can identify that some form of service exists, can be reached, and will impact their health.^2^ The approachability of the health care system is therefore dependent upon both the population's health literacy, knowledge and beliefs related to health and sickness, and the system's transparency, information dissemination, and outreach efforts.

Systems that strive to be approachable must consider the health literacy, knowledge, and beliefs of the populations they serve. Although health literacy shows positive trends at the population level, inequities persist for sub‐populations, including minoritized populations and rural residents.^39^ Existing research on health literacy largely relies on samples of individuals who already have an established connection with the health care system. This approach cannot identify the impact low health literacy has on an individual's interaction with the health care system when individuals have limited or no interactions.^40^ Individuals with low health literacy are more likely to delay care and have difficulty finding providers than those with adequate health literacy.^40^ It is imperative to strategically engage hard‐to‐reach populations before they need to approach the health care system.^41^ To advance health equity, society's actions to advance health literacy must be aligned with the complex factors that affect individual's ability to know that they need care even before they begin the care‐seeking process and then find, understand, and use health information.^42^

AHRQ can generate consensus on the role of health literacy in approachability of health care and fund research that seeks innovative solutions to addressing health literacy before the care‐seeking process even begins.

The bidirectional relationship between the system and the population in need highlights the role of trust and necessitates an evidence‐based approach to building trusting relationships in health care.^43^, ^44^, ^45^, ^46^, ^47^, ^48^, ^49^, ^50^ Trust in health care has declined over the past half‐century and is even lower among Black communities, which have experienced consistent barriers to health care access, disproportionate health care disparities, and overt racism within the health care system.^46^, ^51^ Strategies to engender trust within communities include a commitment to health equity, use of data to measure progress toward equity, reliance on comprehensive needs assessments to inform care, establishment of collaborative partnerships across sectors (e.g., housing and education), planning for care transitions, and facilitation of patient engagement.^52^

Systems can also build trust^10^ by examining institutional policies with an equity lens, establishing accountability frameworks, such as equity scores, auditing curricula and clinical algorithms for erroneous use of race, promoting pipelines for students of color, ensuring leaders have training in equity and antiracism, creating real‐time reporting mechanisms to track and respond to racist behavior, reviewing vendor relationships to support minority‐owned businesses, creating opportunities to build wealth among staff in the workplace, and listening to patients and health professionals of color.^10^ Several organizations, including the American Medical Association (AMA), which developed STEPS for health systems to achieve health equity^53^ and the Association of American Medical Colleges (AAMC), which has a trustworthiness toolkit,^43^ have provided broad guidance.^53^

AHRQ can partner with these organizations to develop and evaluate a comprehensive evidence‐based toolkit for systems seeking to become trustworthy and anti‐racist to advance health equity^43^, ^44^, ^53^ After generating consensus on trustworthy practices of health care systems, AHRQ can promote funding that not only tests these principles but also measures trustworthiness of the system, as opposed to a focus on individual‐level trust.^44^

Researchers have tackled issues of transparency, health literacy, and trust by employing community‐engaged research strategies. They demonstrated that these factors can be reinforced through partnerships that promote co‐learning and co‐production,^54^, ^55^ where the community has input into the best use of resources to serve their needs and is involved in all aspects of research or health care services design and implementation.^56^ Another promising strategy is partnering with trusted entities within the community (e.g., faith‐based organizations or barbershops), which can not only bolster research efforts but also facilitate initial access to care and improve chronic disease management.^48^, ^57^

AHRQ can spearhead national efforts to provide training to researchers in co‐production, a community‐engaged research mechanism that promotes sharing of power and an asset‐based approach. AHRQ can also support funding that promotes measurement of the impact of community engagement efforts, including novel strategies such as use of social media for information dissemination to address health literacy and transparency, monthly community grand rounds, partnerships with non‐clinical entities, or even employing the tenets of being an anchor institution (an institution that hires, purchases, invests, and volunteers locally).

## 4 ACCEPTABILITY

Access to care varies by *acceptability* of care in the Levesque et al. framework, or social and cultural factors that dictate provider attitudes about acceptable characteristics of patients, as well as the patients' attitudes about acceptable characteristics of their providers.^2^, ^36^ Racially/ethnically minoritized patients not only report less satisfaction with care compared to their White counterparts,^58^ but also more frequently describe experiences of disrespect within the health care system.^59^, ^60^, ^61^ Such negative perceptions and experiences may also be exacerbated among patients with overlapping social identities (e.g., Black transgender patient and Spanish‐speaking patient with a disability),^3^, ^4^, ^58^, ^59^, ^60^, ^61^, ^62^, ^63^, ^64^ resulting in an accumulation of discrimination.

While several aspects of care determine acceptability, the Summit focused on the confluences of racism and its relationship to *professional values* and the *racial/ethnic diversity of providers*, both of which reflect the broader beliefs and values of the health system. Structural racism is the totality of ways institutions and systems interact to assert racist practices, policies, and beliefs about racialized groups.^16^ It is produced and reproduced by laws, rules, and practices embedded in the economic system and in norms that influence professional values.^5^, ^6^, ^8^, ^16^ Therefore, confronting racism necessitates altering individual attitudes as well as fundamental change in the racist policies and institutions that are root cause of U.S. racial hierarchy and resulting health inequities.^8^ Racism is not experienced in isolation from other forms of marginalization. Intersectionality, increasingly recognized as an important tool for health equity research, considers individuals with multiple marginalized identities who are subject to interactive mechanisms of oppression.^3^, ^4^, ^58^, ^62^, ^63^, ^64^, ^65^

There is limited consensus on best practices in education and training for providers to gain competency in structural racism, racial biases, intersectionality, and how these biases contribute to downstream health inequities. Research demonstrates that health systems and the providers within the systems espouse egalitarian views but concurrently manifest racial biases, which are associated with differential treatment, lower patient satisfaction, and ultimately delays in seeking care.^5^, ^8^, ^48^, ^66^, ^67^, ^68^, ^69^ The greatest health inequities are found between Black and White patients.^70^ Black adults are more likely than White adults to experience a provider not believing them and refusing them a treatment, test, or medication they thought they needed.^51^ The entities that work to address these biases often work in silos, but no single entity can address the issue alone. The AMA has led multiple efforts,^71^, ^72^ as have CMS,^14^ the AAMC,^73^ the U.S. Health and Human Services with their Equity Action Plan,^18^ among others, to train health care providers and patients on mitigating racism in health care.^74^

AHRQ's focus on training and care delivery creates an opportunity to lead and partner with professional associations (e.g., AAMC and Accreditation Council for Graduate Medical Education (ACGME)) to champion the importance of using evidence‐based tools. This may include the development of health equity toolkits, standardized provider trainings or curricula, and other tools that can support care that better meets the needs of diverse patient populations—thereby improving equitable access and quality of care. One focus for this work can be ensuring continued evaluation of existing programs over time and using an implementation science lens, which will help determine best practices for different populations and contexts.

Targeting bias is necessary but not sufficient, as marginalized populations are underrepresented in the current and projected health care workforce.^75^ Race‐ or gender‐identity‐concordant relationships between providers and their patients result in higher patient satisfaction and better health outcomes.^59^, ^60^, ^61^ Therefore, diversifying the workforce can help advance equity in health care acceptability. A burgeoning approach to addressing diversity among physicians is the use of holistic review during recruitment, which goes beyond academic metrics and considers the lived experience of the applicant and their potential contributions to promote workforce diversity.^76^, ^77^

AHRQ can no longer ignore the underlying issue of the lack of diversity in the health care workforce across all levels.^76^, ^77^ Holistic review is not a standardized practice across the nation, and some programs describe institutional barriers to conducting such reviews. AHRQ can partner with the AAMC and ACGME to create metrics for both holistic review and retention, evaluate the use and impact of these metrics in varied institutional settings, and ultimately create an evidence‐based standard to improve diverse recruitment and retention. Although there is evidence supporting pipeline programs, few evaluate long‐term outcomes. AHRQ is uniquely positioned to partner with HRSA and fund multi‐year analyses focused on the long‐term outcomes of pipeline programs that invest in the communities with the highest need.

## 5 AVAILABILITY AND ACCOMMODATION

Availability and accommodation in the Levesque et al. framework encompass the physical existence of health resources and facilities that can be accessed in a timely manner and with sufficient capacity.^2^, ^36^, ^38^ The health care system's ability to *reach* patients equitably is dependent on the *availability* of services and whether those services are *accommodating* to the entire population.^2^, ^34^, ^36^, ^38^, ^78^, ^79^ How services are structured and designed influences whether they accommodate the needs of the population they aim to serve.

Several factors, independently and interdependently, influence the availability of and accommodating nature of services and influence observed health inequities. These factors include the existence and geographic distribution of facilities and providers, the modality of delivery (e.g., in‐person, telemedicine, or home visits), who delivers that care (e.g., health care professionals, navigators, or community health workers), and when those services are offered (e.g., after hours or weekends), availability of language services, and accessibility accommodations for the elderly and people with disabilities.^2^, ^36^, ^38^, ^80^, ^81^

The Summit discussion specifically highlighted the availability and accommodation information gaps for individuals with limited English proficiency (LEP), substance use disorders (SUDs), and disabilities, as well as the elderly and socioeconomically disadvantaged patients unable to seek care during traditional hours. LEP contributes to significant barriers to accessing care.^18^, ^80^, ^81^, ^82^, ^83^, ^84^, ^85^ The U.S. Department of Health and Human Services' Office of Minority Health (OMH) funded over $4 million across 11 sites to implement and evaluate strategies to enhance language access services.^82^ Rather than structure recommendations around this important issue at this time, AHRQ can partner with OMH to disseminate best practices once the projects are evaluated. The workgroup agreed to focus on ameliorating supply‐side constraints that impact all these issues, in particular workforce issues.

Health care provider shortages were an overarching concern for the workgroup, including shortages of physicians, nurses, and community health center staff.^86^ The workgroup consensus was to focus on the issue of physician shortage, as it is a prominent and primary driver for inequitable availability of health care services. The AAMC projects that by 2034, the U.S. will experience a physician shortage of up to 48,000 primary care physicians and 77,100 non‐primary specialists.^87^ Such shortages, although concerning for all populations, have disproportionate impacts on Black, Hispanic, and indigenous populations, as well as those living in rural areas.^75^, ^87^, ^88^, ^89^ Furthermore, the AAMC reports that if underserved populations had the same health care use patterns as populations with greater access to care, the nation would need an additional 184,000 physicians.^87^ Living in areas with a greater density of primary care providers is associated with lower mortality rates and better population health outcomes, highlighting the urgent need to address this shortage.^22^

Physician shortages cannot be discussed without the inclusion of burnout,^90^, ^91^, ^92^ exacerbated by the COVID‐19 pandemic,^93^, ^94^, ^95^ with 62.8% of physicians reporting at least one manifestation of burnout in 2021.^95^ Nearly 25% of providers consider switching careers,^96^, ^97^ and over 1 in 5 physicians report experiencing a patient or their family refusing to allow them to provide care due to personal attributes of the physician.^98^ Experiencing discrimination or mistreatment is associated with a higher likelihood of experiencing burnout,^98^ necessitating the importance of addressing the impact of racism on burnout.^99^ Although evidence‐based programming to combat burnout exists, many of these programs focus on individual strategies, do not focus on systemic inequalities, and do not use rigorous designs that incorporate a health equity lens.

AHRQ can fund research that develops and evaluates tools focused on combating system‐ or institutional‐level factors associated with burnout, such as poor workflow, high levels of clerical responsibilities, and poor configuration of the electronic health record. Such research can aim to go beyond preventing or mitigating burnout toward enhancing physician well‐being and fulfillment through the creation and utilization of measures that assess constructs of physician “thriving.” The complexity of provider discrimination, its impact on burnout, and ways to retain the marginalized health care workforce must be given more attention. AHRQ can solicit proposals with a focus on minoritized groups' experiences and with novel interventions that simultaneously concentrate on reducing discrimination and improving physician well‐being.

## 6 AFFORDABILITY

Affordability reflects the relationships among prices of services, patients' ability to pay, and existing health insurance.^36^ Levesque's model describes three types of costs associated with use of health care services: *direct costs* (e.g., out‐of‐pocket costs), *indirect costs* (e.g., transportation costs), and *opportunity costs* (e.g., forgone wages).^2^ High out‐of‐pocket costs and medical debt are associated with unmet needs and delayed or forgone care, particularly for marginalized populations.^12^, ^100^ The juxtaposition of high health care costs and worsening income and wealth inequality that disproportionately impacts marginalized populations, disabled individuals, and non‐cisgender populations underscores the critical need to address barriers to affordable care for all.^15^

Direct costs can be reduced by both securing health insurance and maintaining it. Having coverage has been associated with reductions in cost‐related barriers to care, financial stress, maternal and infant mortality, and increases in preventive care.^12^, ^101^, ^102^, ^103^, ^104^ Disparities exist in access to health insurance coverage among AI/AN, Latine, and Black populations,^28^, ^29^ as well as for undocumented immigrants, those in rural communities, and transgender individuals, which in turn hinders access to care.^105^ Lack of insurance is associated with lower use of preventive services,^106^, ^107^ higher disability rates, poorer health outcomes, and being sicker when diagnosed.^34^, ^78^

Even among insured individuals, increases in patient cost‐sharing have eroded affordability of care and impacted access. The steady growth in High Deductible Health Plans raises concerns about cost‐related barriers to care for marginalized populations who tend to not have Health Savings Accounts. Coverage exclusions that leave out certain services or providers can have downstream impacts on access to care and health.

The Affordable Care Act (ACA) coverage expansions and COVID‐related emergency policies helped reduce uninsured rates to a historic 8% low in 2022.^108^ Medicaid expansions facilitate access to care and reduce disparities in uninsured rates across socioeconomic status, race, age, and rurality, yet inequities persist.^30^, ^109^ The remaining 10 states that have opted not to expand Medicaid are largely located in the Southeast with Black populations that exceed the national average, leaving 1.9 million low‐income adults without coverage.^110^ It is important to note that in these 10 states, adults in the coverage gap are disproportionately racially/ethnically marginalized populations, and have incomes above their state's eligibility for Medicaid but below the poverty threshold, making them ineligible for subsidies on the ACA Marketplace.^110^ However, even those insured adults who are between 100% and 138% of the poverty level face out‐of‐pocket costs that reduce their access to care, which would be lower if they had Medicaid coverage. Implementation of the ACA's antidiscrimination rule could impact coverage.^30^, ^66^, ^102^, ^103^, ^104^, ^109^, ^110^, ^111^, ^112^, ^113^, ^114^, ^115^, ^116^, ^117^, ^118^, ^119^

Inequities are a universal challenge, and no one entity can address health inequities on its own. Multi‐stakeholder collaborations are essential. AHRQ can partner with private entities that have initiatives on unified industry standards in measurement, including the Blue Cross Blue Shield Association^15^ and can collaborate with the Centers for Disease Control and Prevention (CDC) to promote consistent data collection using metrics that incorporate the OMH granular categories of race and ethnicity to assess structural racism and experiences of discrimination when accessing insurance and standardized measures of generosity of insurance coverage (including cost‐sharing, covered services, and provider networks) to ensure equitable access to affordable care.^15^, ^80^, ^120^

Minimizing enrollees' churn, or temporary losses of Medicaid or other insurance coverage due to changes in eligibility, is also critical to reduce costs for disadvantaged populations.^121^, ^122^ Many enrollees experience churn due to administrative barriers, such as determining if they are eligible and completing complicated applications, which exacerbates inequities as churn is associated with access barriers.^121^ During the COVID‐19 Public Health Emergency (PHE), states were required to freeze Medicaid eligibility to continue receiving federal matching funds.^113^ Following the end of the PHE in 2023, it is estimated that 14–15 million individuals could lose Medicaid.^113^, ^122^

Many administrative barriers are the product of policy choices that reflect racism. The ACA implemented provisions intended to reduce administrative burdens for Medicaid enrollees, and the Biden Administration and Congress have taken a range of actions to strengthen these provisions and extend them to other populations.^123^ States are also extending continuous eligibility periods for certain categories of enrollees, including children and pregnant women in the postpartum period. These policy changes could help address racial inequities in maternal mortality for Black and AI/AN women, particularly at the intersections of low SES and rurality.^101^, ^121^, ^124^ It will be critical to evaluate how states' unwinding processes and redetermination requirements will impact equity of access to coverage.^113^, ^122^

The continuous Medicaid enrollment during the pandemic substantially reduced churn,^122^ providing an opportunity to evaluate the impact of reduced interruptions in coverage. AHRQ can partner with CMS to fund priority studies that evaluate the cost of the coverage protections during the pandemic, the derived benefits for the Medicaid population, and costs to the states and federal government of permanently removing the eligibility barriers. AHRQ can also fund initiatives to collect data on and explore the impacts of federal and state efforts to reduce administrative burdens for enrollees and identify further improvements in the ACA to facilitate coverage expansions.

## 7 APPROPRIATENESS

Appropriateness relates to the fit between services and needs of the patient, community, and population.^2^ The construct of appropriateness is broad, and encompasses which services are provided, how the services are provided, and, importantly, the ability to participate in health care decision‐making. Each has been found independently to be associated with inequities in access and quality of care.^2^, ^38^ Inequity in quality of care for racially/ethnically marginalized populations serves as a barrier to appropriateness of care,^125^ while patient's ability to participate in decision making has been shown to be an important factor in ensuring access to appropriate care.^126^, ^127^

Providers have increasingly used practice guidelines and diagnostic algorithms that adjust for race or ethnicity, which can propagate race‐based medicine.^21^ De‐implementation of low‐value care for minority populations and race‐based medicine can level the playing field for appropriateness of care.^21^, ^128^ For example, an algorithm that calculates a lower rate of success for vaginal birth after caesarean (VBAC) informed by race/ethnicity could exacerbate the already existing disparities in maternal mortality.^21^

Efforts are underway in the health care system to de‐implement non‐evidence‐based practices and correct for race/ethnicity in algorithms. AHRQ can fund initiatives to quantify the impact of de‐implementation on minority populations.

Empowering patients with their own data helps them make more appropriate, informed decisions about their health.^15^ AHRQ has been working to fill important gaps relating to digital health.^129^ These areas of work continue to be paramount and important targets, but they must explicitly focus on reducing inequities.^39^ Patient clinical decision support can operationalize tenets of a shared decision‐making process and patient‐centered care and should be developed with a health equity lens.^130^ Prior research has highlighted that disadvantaged groups may benefit more from shared decision‐making tools than other groups with higher health literacy.^131^ AHRQ also recently released the “Question Builder” tool, which helps patients and caregivers prepare for an appointment by organizing questions and health information to share with the clinical team.^132^

Intersectionality and digital health inequities are emerging fields of research where AHRQ can serve as the trailblazer in systematically assessing the existing research and conceptualizing best practices for digital health decision‐making tools.

## 8 DISCUSSION

AHRQ organized a working group to develop recommendations for strategic investments in improving access to care with a health equity lens. These recommendations are not without limitations. With more time and resources, the workgroup would expand the dialog beyond the physician focus and address other important issues like LEP, telehealth, transportation, and behavioral, mental, and reproductive health. The workgroup had an extensive conversation around cost‐sharing and coverage gaps in care, but struggled to identify recommendations that were within AHRQ's scope.

AHRQ's mission is to produce scientific evidence that makes health care safer, higher quality, more accessible, equitable, and affordable.^133^ AHRQ achieves its science and implementation missions through partnerships with federal, state, tribal, and local agencies; with non‐governmental groups and professional associations; and with health care systems and industry. AHRQ can adjust these characteristics and impact access in a meaningful way. Inequities in access, health, and disease across populations are like any other diagnostic or therapeutic challenge. Progress will be made when there is common acceptance that inequities in access, health, and disease arise from a disease process rooted in social experience and infrastructure rather than DNA.^70^ Detailed strategies to combat racism and data on inequities in access to care are essential steps in addressing the issue and formulating effective solutions. These recommendations provide an innovative approach for AHRQ to leverage existing resources to build collaborative partnerships and ultimately push the field forward to improve equity in access to health care.

## Supporting information


**Appendix S1.** Supporting Information.
