# Supplementary material for: Eliminating health care inequities through strengthening access to care
Source: Health Serv Res. 2023 Nov 28;58(Suppl 3):300–10. doi: 10.1111/1475-6773.14202 (PMC10684044; doi:10.1111/1475-6773.14202)
Supplement: Supplementary file 1 — Appendix S1. Supporting Information. [file HESR-58-300-s001.docx]

## Overview Paper HSR Supplement (AHRQ Equity)

In 2021, the Agency for Healthcare Research and Quality (AHRQ) launched a multiphase stakeholder engaged process to develop an AHRQ Equity Agenda and Action Plan to guide setting priorities to advance health equity within our nation’s health care delivery systems.

December 2021 AHRQ convened a stakeholder planning meeting that included more than 50 diverse stakeholders and users of AHRQ research, data, and tools. Participants included healthcare systems leaders, health equity researchers, and patient and family advocates. The meeting was an important initial step to plan an AHRQ Health Equity Summit (noted as Summit, hereafter). The group discussed the scope, goals, key research and action domains for AHRQ priority setting, and strategies to center principles of diversity, equity, inclusiveness and accessibility within the design and structure of the Summit. Additionally, the planning meeting participants provided AHRQ with feedback on an Equity Research and Action Framework that would be used to guide subsequent phases of work on the AHRQ Equity Research and Action Plan.

Between the planning meeting and Summit, AHRQ leveraged the planning meeting feedback to finalize the Framework, outlining the core research and action domains. AHRQ established five expert writing teams to co-author invited papers aligned with the Framework: *Healthcare Delivery Systems; Payment; Implementation Science; Social Determinants of Health; and Access to Care*. The teams were tasked with conducting evidence-based narrative reviews focused on the intersection of health equity and their assigned domain. AHRQ took a pragmatic approach to paper development. Rather than request formal systematic reviews, AHRQ allowed each paper team to tailor its approach to their topic, state of the literature for that topic, and the rapid project timeline. The existence of numerous reviews in the scientific literature that could be leveraged by author teams contributed to this decision. The writing groups were asked within the narrative reviews to identify research needs/gaps that if filled could accelerate equity and to make specific recommendations on how AHRQ could serve as a catalyst to drive more equitable healthcare. To ensure experiences and voices of diverse stakeholders could be integrated into the invited papers, prior to the Summit the author teams shared an outline of their papers and a set of structured questions that would be used to guide a facilitated group discussion at the Summit.

The 2-day virtual Summit took place July 27-28, 2022. All sessions were recorded, and participants were prompted in Zoom to provide consent. The Summit was focused on establishing a community of belonging to co-create and develop a common understanding of strategies needed to advance equity within the healthcare system and opportunities for AHRQ to drive progress. Specifically, the goals of the Summit included: (1) development of a common understanding and language to describe health equity in the context of AHRQ’s vision and mission; (2) exploration of shared experience of stakeholders with regard to the lingering impact of structural racism on the healthcare delivery system and implications for system leaders focused on healthcare delivery system performance improvement; and (3) empowering stakeholders to serve as change agents committed to improving equity in all spheres of influence including participation in an intentional, structured, co-creation process to inform the AHRQ Equity Agenda and Action Plan for advancing health equity.

AHRQ utilized participatory full-group and small-breakout sessions to achieve Summit goals. The shared full-group community-building sessions bridged to smaller break-out sessions, each focusing respectively on one of the five key research and action domains. Each small-group participant had received the initial paper outline and questions prior to the Summit to facilitate eliciting feedback and active engagement. One paper author and a skilled equity facilitator co-facilitated each small group session and the groups utilized Jamboard, a real-time, dynamic, collaborative tool. These processes were designed to prioritize empowered and equitable participation and ensure all voices and contributions from the diverse stakeholder community were captured for later use by the author teams after the Summit.

After the Summit, AHRQ provided each author team with recordings from the breakout sessions and images of individual Jamboards to inform the papers. Each team tailored methods/processes for their review to their specific domain, which is briefly described in each respective paper. The authors met with AHRQ within one month of the Summit to discuss updates to their outlines based on stakeholder input and resolve potential areas of overlap. Finally, the respective writing groups worked independently to produce and submit papers.
